# Supplementary material for: Novel Insights into Changes in Gene Expression within the Hypothalamus in Two Asthma Mouse Models: A Transcriptomic Lung–Brain Axis Study
Source: Int J Mol Sci. 2024 Jul 5;25(13):7391. doi: 10.3390/ijms25137391 (PMC11242700; doi:10.3390/ijms25137391)
Supplement: Supplementary file 1 [file ijms-25-07391-s001.zip › ijms-3017601-supplementary.pdf]

Supplementary Materials

A. Figures

- Supplementary Figure S1 (Confirmation of Lung Inflammation)
- Supplementary Figure S2 (Validation of the microdissection: Marker genes detected in our study)
- Supplementary Figure S3 (Gene expression changes in the hypothalamus response to asthma)
- Supplementary Figure S4 (Heatmap of the top 50 genes showing the effect of LPS- and OVA-induced asthma on the expression of single genes)
- Supplementary Figures S5 and S6 (A detailed description of the significant gene sets)
- Supplementary Figure S7 (Top 20 REACTOME gene sets as ranked by magnitude of effect of size after filtering for FDR<0.05 in response to LPS and OVA (mitch, FDR < 0.05).
- Supplementary Figure S8 (Effect of LPS-and OVA-asthma induction (two combined groups).
- Supplementary Figure S9 (Heatmap for top-50 discordant dysregulated gene)
- Supplementary Figure S10 (Timeline of the two asthma models used in this study)
- Supplementary Figure S11 (Brain microdissection)

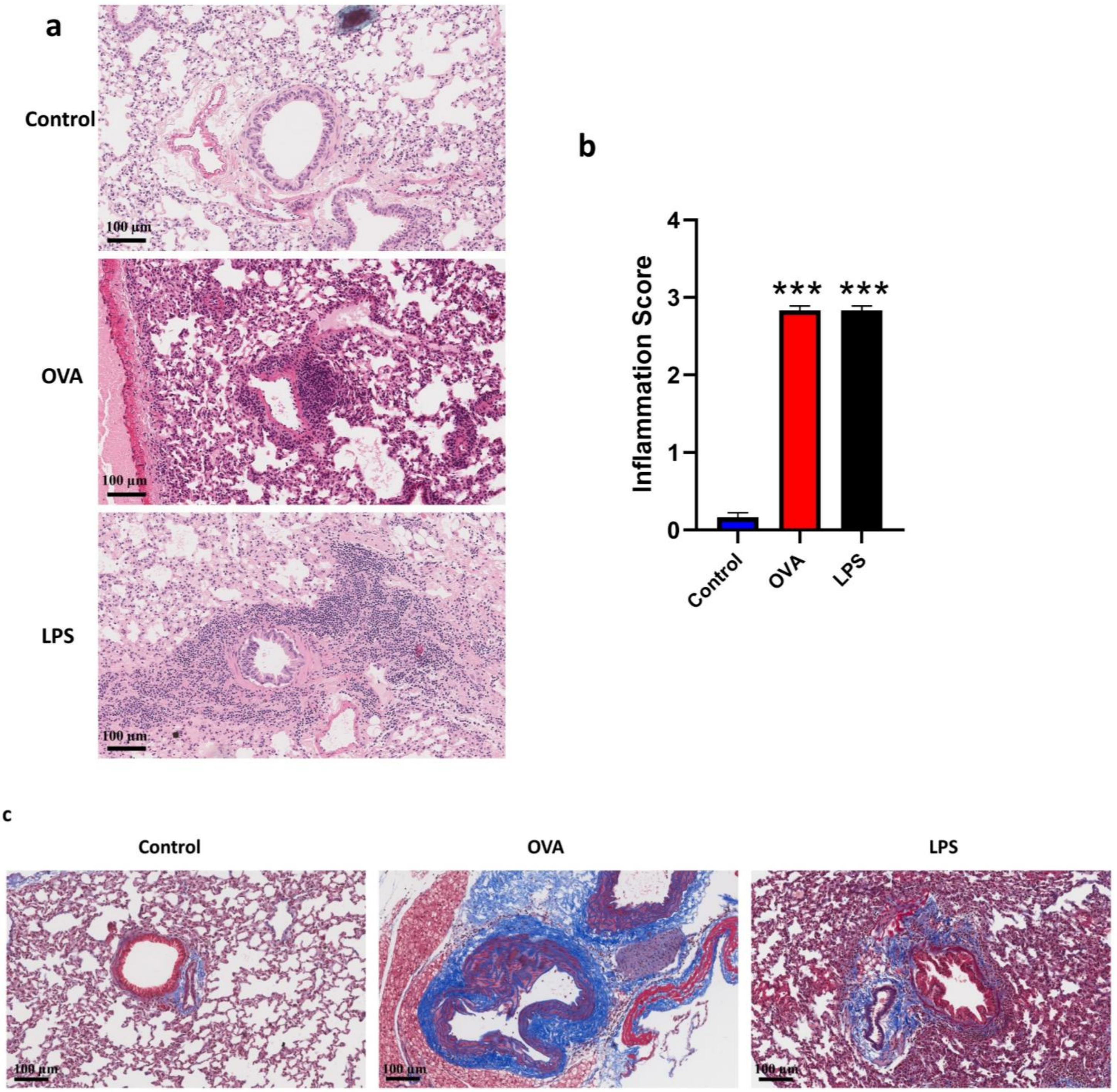

Supplementary Figure S1. Histological validation of lung inflammation within our asthma models.

(**a**) Representative examples of haematoxylin and eosin-stained mouse lung tissue, whereby typica asthma features were observed in both OVA and LPS-challenged group as compared to the control group, as indicated by the excessive infiltration of inflammatory cells surrounding the bronchioles and blood vessels. (**b**) Quantification of lung inflammation scores, using haematoxylin and eosin stain (n=6 per group), bars represent mean ± SEM. ***$p<0.001$ (one-way ANOVA). (**c**) Representative examples of Masson's trichrome stain, not quantified, whereby blue staining indicates fibrosis / collagen deposition. Scale bar 100µm.

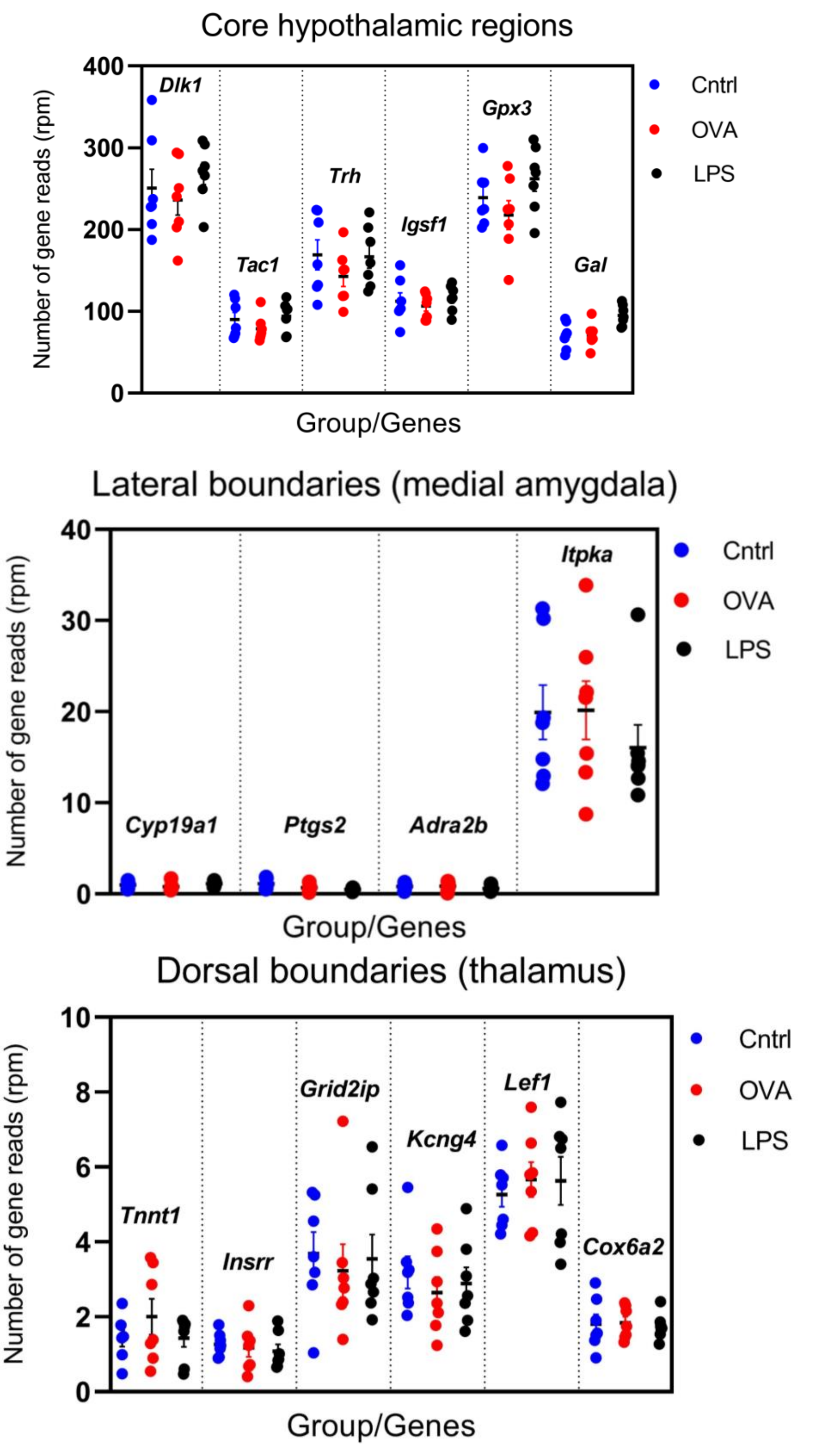

Supplementary Figure S2. Marker genes detected in our study within the hypothalamus, lateral boundaries (medial amygdala), and dorsal boundaries (thalamus).

Data from specific genes was extracted from whole transcriptome data, based on their neuroanatomical distribution (visualised within the Allan Brain Atlas (REF) relative to the collected hypothalamic region. Genes are grouped within their treatments (control, OVA, and LPS; n=7 mice per group) and assessed via nested one-way ANOVA. Abbreviations: *Gpx3*: glutathione peroxidase 3 gene, *Tac1*: tachykinin-1, *Dlk1*: Delta-like 1 homologue, *Trh*: thyrotropin-releasing hormone, *Igsf1*: immunoglobulin superfamily member 1, *Cyp19a1*: aromatase, *Ptgs2*: prostaglandin D2 synthase, *Adra2b*: alpha 2b adrenergic receptor, *Itpka*: Inositol-trisphosphate 3-kinase A, *Tnnt1*: Troponin T1, *Insrr*: insulin receptor-related receptor, *Grid2ip*: glutamate receptor, ionotropic, delta 2 interacting protein 1, *Kcng4*: potassium voltage-gated channel subfamily G member 4, *Lef1*: lymphoid enhancer binding factor 1, *Cox6a2*: cytochrome c oxidase subunit. Seven mice were used.

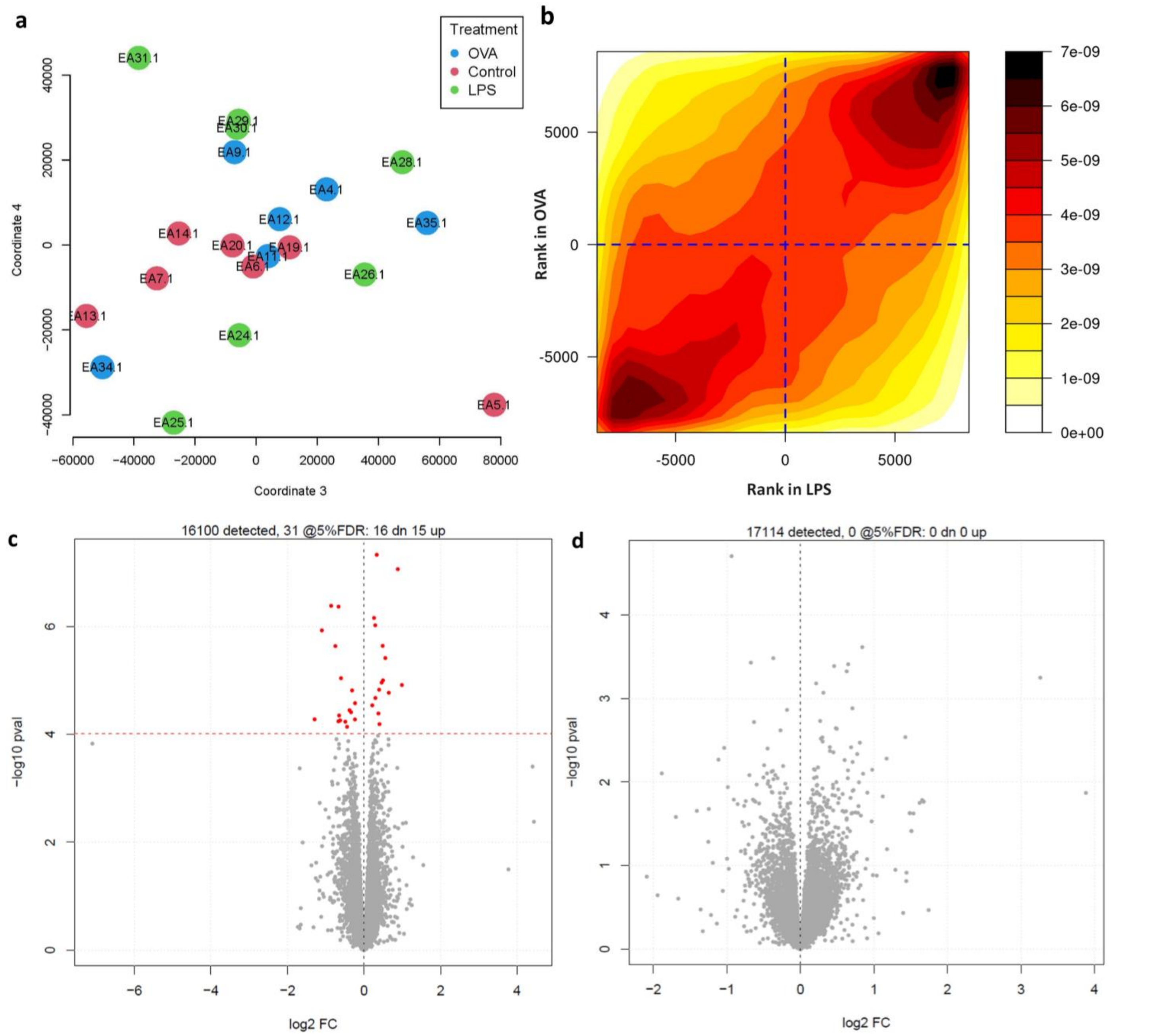

Supplementary Figure S3. Gene expression changes in the hypothalamus response to asthma.

(a) Multidimensional scaling analysis reveals that asthma challenge using LPS and OVA have a modest effect only, relatively few genes are significant between treatment groups. (b) Rank-rank (contour) density plot of gene expression changes following LPS versus OVA treatment, relative to control, showing similar gene expression score ranks in both asthma groups. (c) Volcano plots of $\log_2$ fold change (FC) in LPS-treatment relative to control, and OVA-treatment relative to control (d), versus -log(p-value). The X-axis represents logarithm with base ($-\log_{10}$ p value), while the Y-axis represents the $\log_2$ fold change ($\log_2$ FC). Statistically significant genes are indicated in red. Notably, no significant genes were detected in the OVA-induced group, whereas the LPS-induced group revealed 31 significant single genes (16 downregulated and 15 upregulated). FDR < 0.05.

a

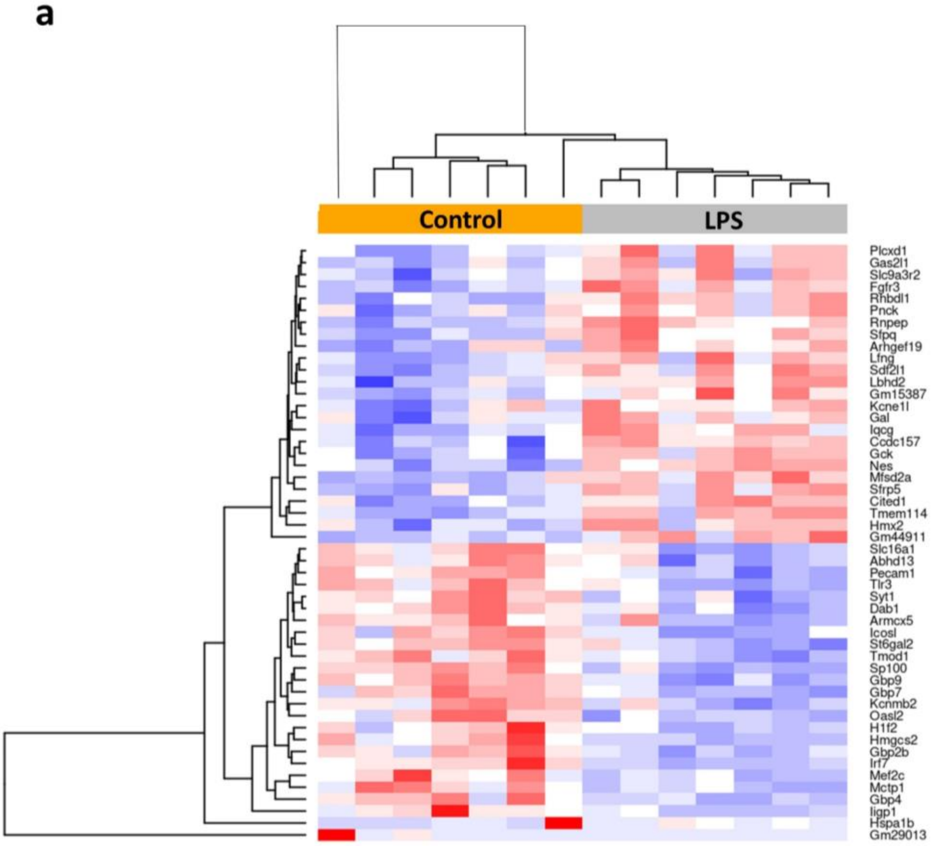

b

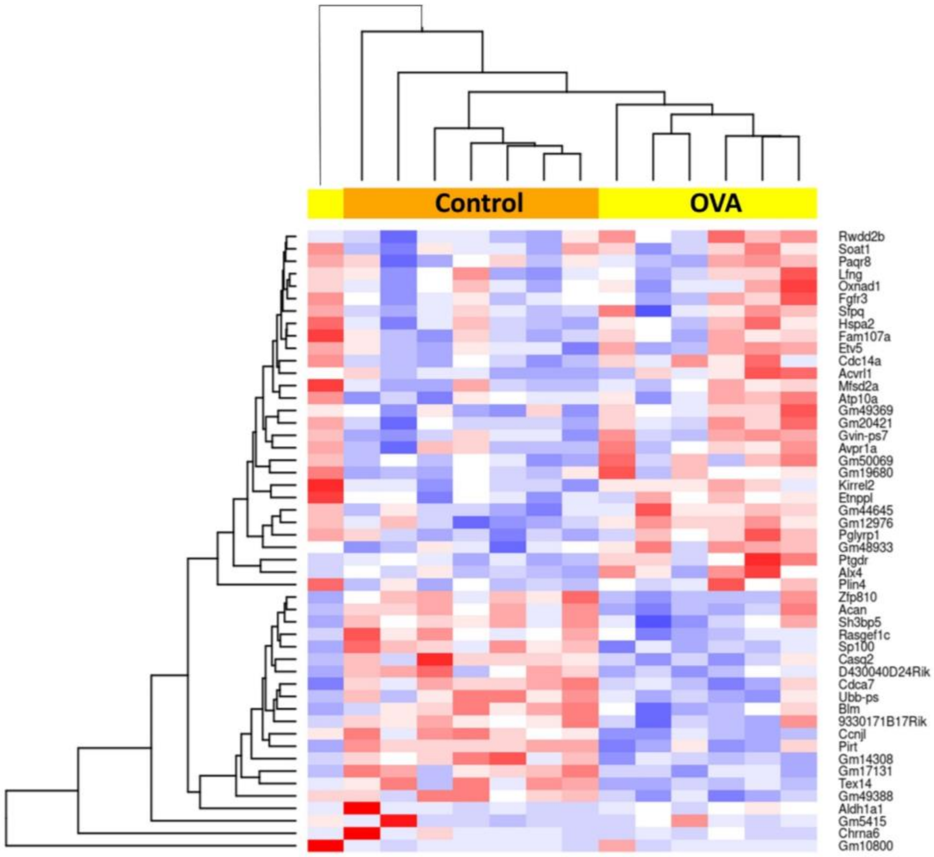

Supplementary Figure S4. Heatmap of the top 50 genes showing the effect of LPS- and OVA-induced asthma on the expression of single genes.

These top 50 genes (ranked by p value / significance) identified following LPS treatment relative to control (a), and OVA treatment relative to control (b).

LPS

- ❑ **Right side (upregulated gene sets)**
  - **Metabolism gene sets (upregulated, yellow highlight).**
  - **Inflammatory gene sets/ Cytokine signalling (black annotated circle):**
    - Asymmetric localization of PCP proteins (signal transduction).
    - Hedgehog ligand biogenesis (Signal transduction).
    - AUF1 (hnRNP D0) binds and destabilizes mRNA (metabolism)
    - Degradation of axin (signal transduction)
    - Cross-presentation of soluble exogenous antigens (endosomes) (Immune system).
    - Dectin 1 mediated non canonical (NF-KB pathway)
    - Stabilizaton of p53 (cell cycle)
    - Negative regulation of notch 4 signalling (signal transduction).
    - Mito protein import (protein localization).
    - Metabolism of polyamines (M).
    - SCF(Skp2)-mediated degradation of p27/p21 (Cell cycle).
    - * Interleukin 6 signalling.
    - * Interferon signalling.
    - TNFR1 induced NF Kappa B signalling pathway .
    - Cytoprotection by HMXO1.
  - **Cellular response to stress (grey highlight):**

  * Cellular response to hypoxia
  * Regulation of HMOX1expression and activity

- ❑ **Left Side (down regulated gene sets)**
  - Neuronal and synapse signalling (orange arrows, blue highlight).
  - Homeostasis signalling (green annotated circles).

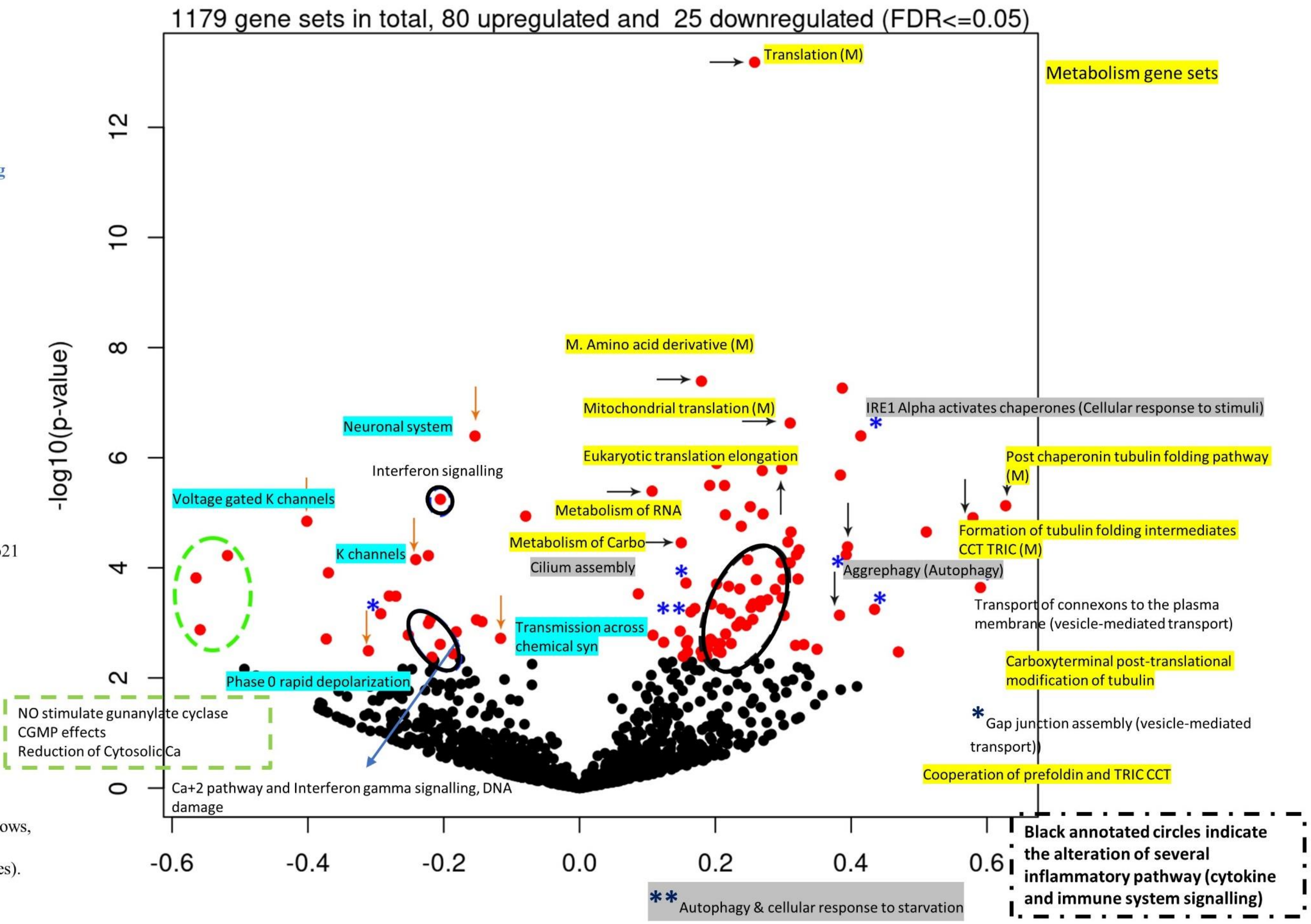

Supplementary Figure S5. Volcano plot of gene set enrichments analysis for the LPS-treated group showing the significantly altered gene sets (GSEA-P,

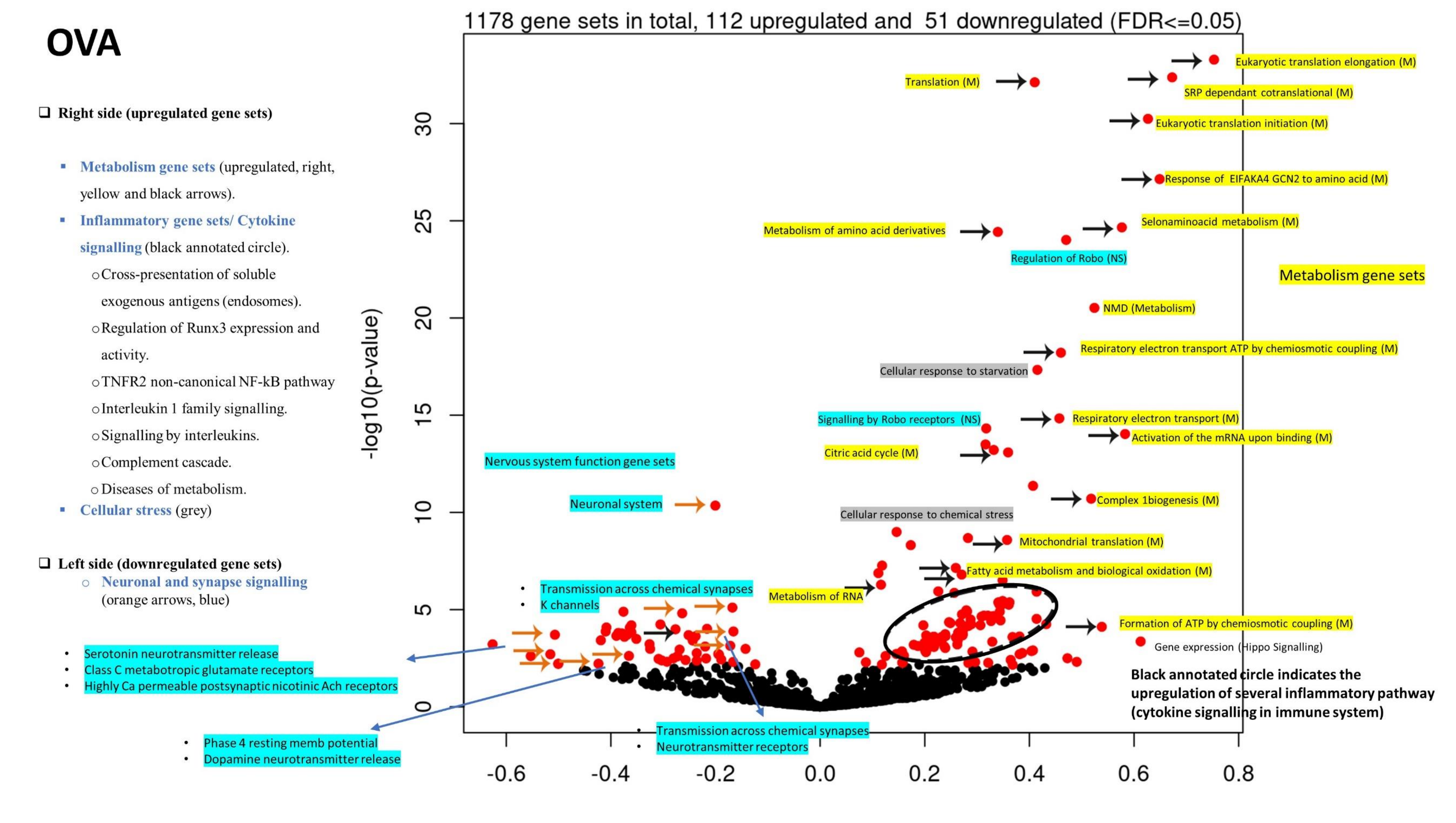

Supplementary Figure S6. Volcano plot of gene set enrichments analysis for the OVA-treated group showing the significantly altered gene sets (GSEA-P, FDR

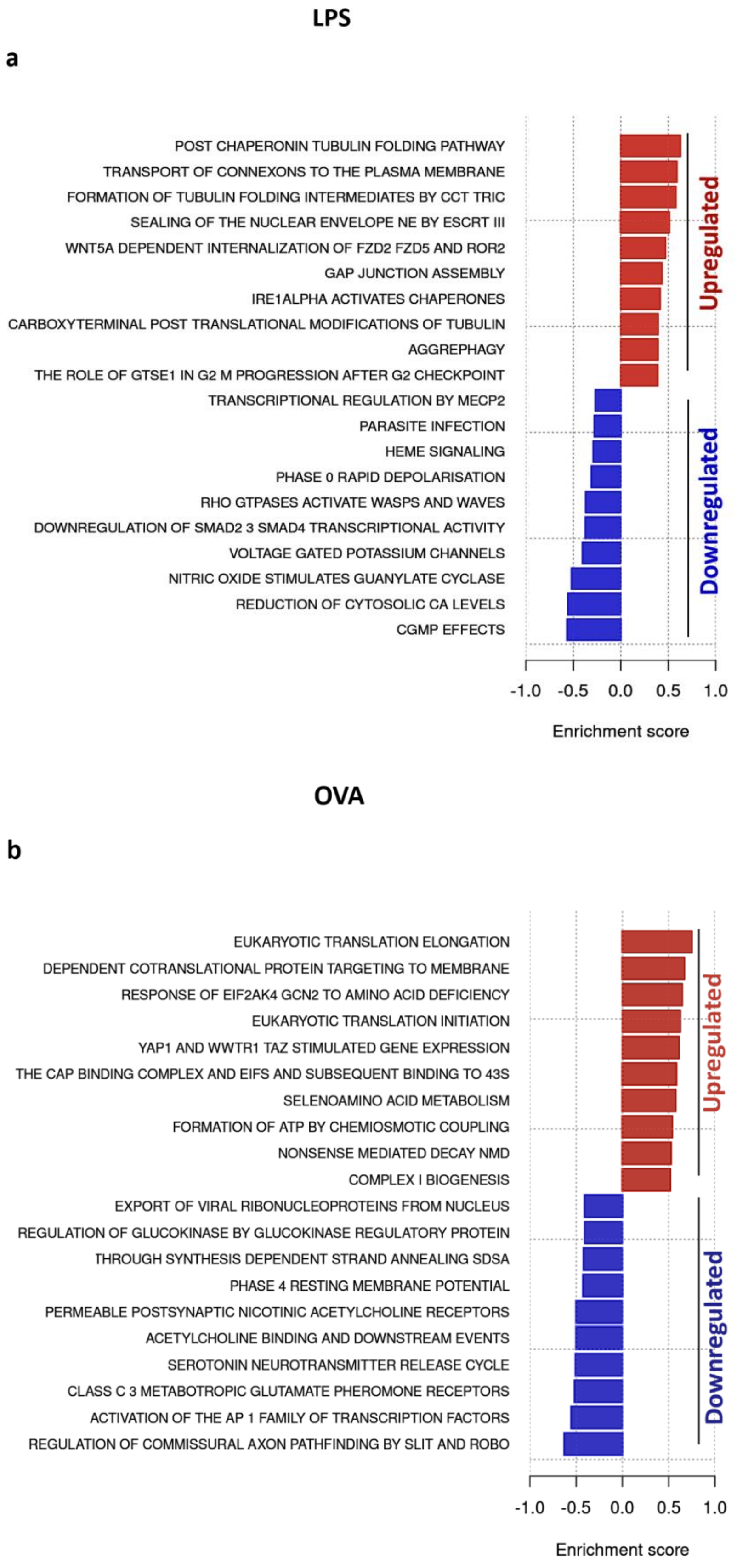

Supplementary Figure S7. Top 20 REACTOME gene sets as ranked by magnitude of effect of size after filtering for FDR<0.05 in response to LPS and OVA (mitch, FDR < 0.05).

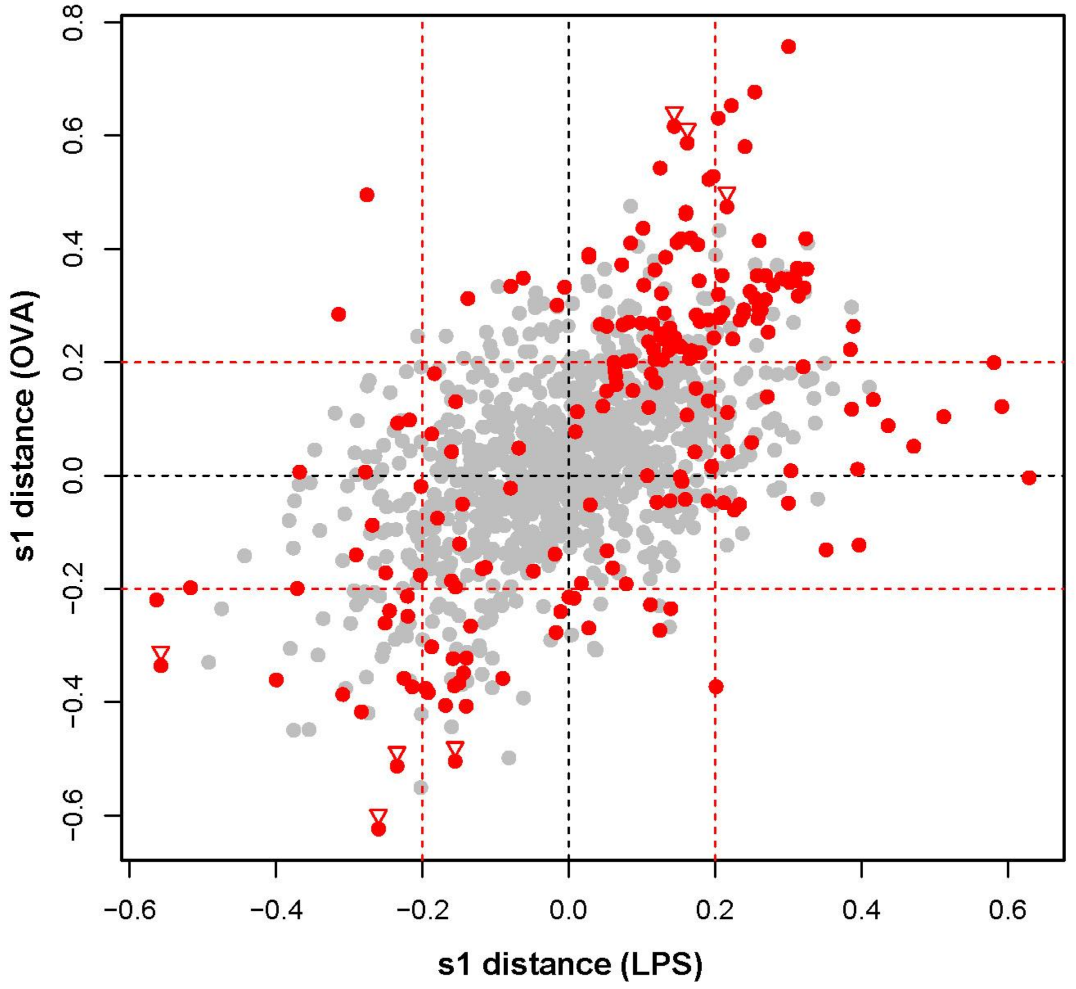

Supplementary Figure S8. Effect of LPS-and OVA-asthma induction (two combined groups).

Scatter plot showing the joint enrichment of all REACTOME gene sets in the two-dimensional space, where the axes represent the enrichment scores for the significant gene sets from mitch FDR<0.05 shown in red and the non-significant gene sets are shown in grey showing an overall concordant pattern. Although a cluster Selected gene sets are indicated by arrows, the

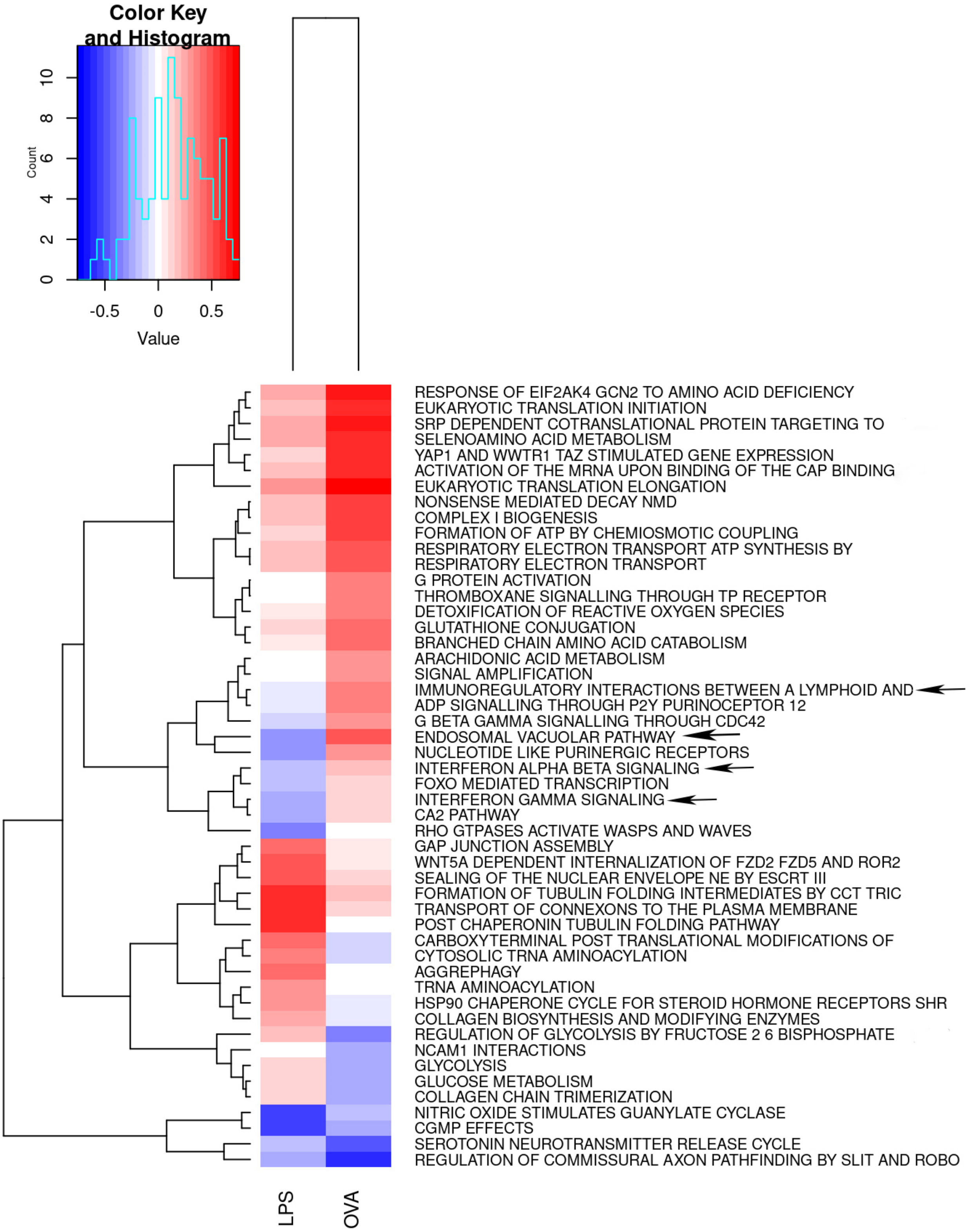

Supplementary Figure S9. Heatmap for top-50 discordant dysregulated gene sets by magnitude of change in asthma relative to the control group. Upregulated and downregulated gene sets are indicated in red and blue, respectively. Among the top 50 dysregulated gene sets, 19 were categorized under metabolism, 5 related to haemostasis, 6 to signal transduction (including Wnt/B-Catenin and GPCR signalling), 3 involved in cellular response to stress, 2 in Hippo and Foxo-mediated signalling, 2 in collagen formation, and 2 in gap junction trafficking. Interestingly, 4 gene sets were found to be directly involved in immune system signalling (black arrows).

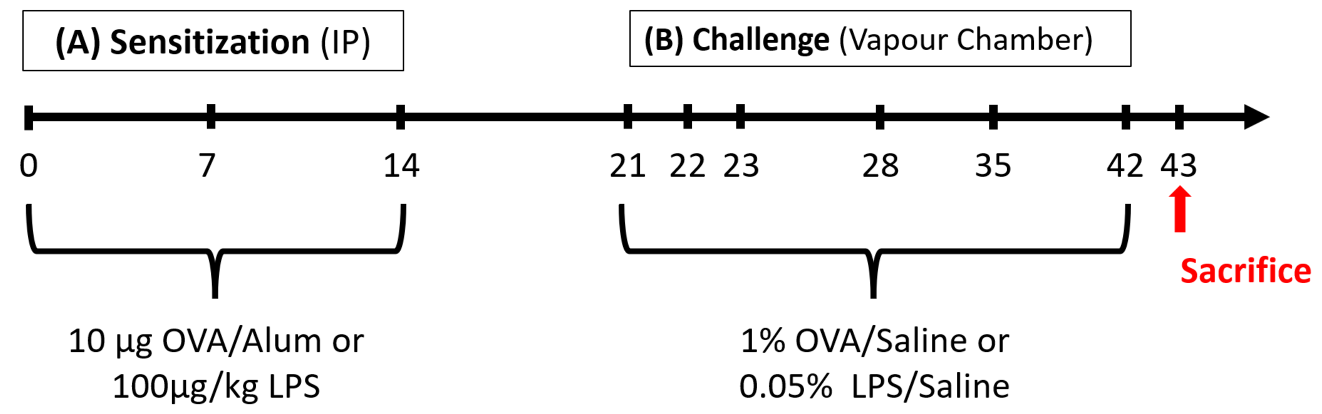

Supplementary Figure S10. Timeline of the two asthma models used in this study.

(A) Intraperitoneal sensitization of OVA (10µg chicken egg with 1% alum adjuvant) or LPS (100µg/kg, E. coli, serotype 026: B6). (B) Challenge of LPS (0.05%, wt/vol) or OVA (1%, wt/vol) in vapour chambers at a rate of ≥0.2ml/min, droplet size <3.3 µm, 20min per day for 3 consecutive days, followed by once/week for 3 additional consecutive weeks. At the end of the experiment mice were culled, then lung and brain samples were

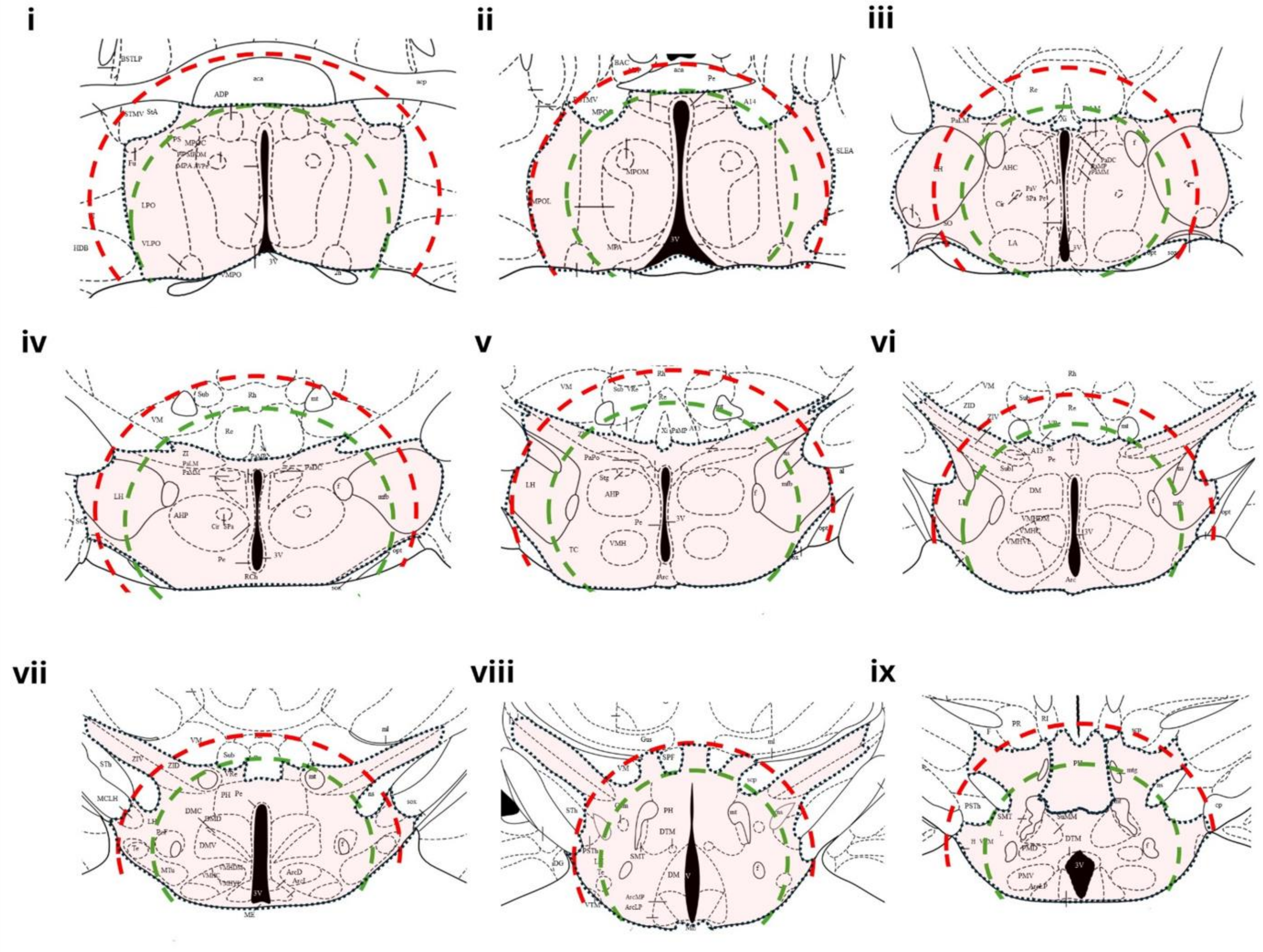

Supplementary Figure 11. Schematic reconstructions of the hypothalamic area collected.

Tissue within the green area was reliably collected in almost all samples whereas region outside of the red area was reliably excluded in almost all samples, and the region in between was somewhat variably collected (bregma 0.14mm to -2.54mm). The areas that were consistently and less consistently collected, indicated by the green-dashed and red-dashed annotations, respectively. The original boundaries of the hypothalamus are defined by the red-shadowed background.

B. Tables

Supplementary Table 1: Top gene sets differences in the hypothalamus for LPS and OVA versus control

Gene set	Set Size	p.adjut MANOVA	s2.distance	s1.distance (LPS)	s1.distance (OVA)	p.LPS	p.OVA
EUKARYOTIC TRANSLATION ELONGATION	87	2.06e-30	0.814	0.3000	0.75700	1.29e-06	2.29e-34
SRP DEPENDENT COTRANSLATIONAL PROTEIN TARGETING TO MEMBRANE	106	9.37e-30	0.723	0.2540	0.67700	6.28e-06	1.71e-33
RESPONSE OF EIF2AK4 GCN2 TO AMINO ACID DEFICIENCY	95	7.97e-25	0.690	0.2220	0.65300	1.83e-04	3.37e-28
REGULATION OF COMMISSURAL AXON PATHFINDING BY SLIT AND ROBO	10	2.11e-02	0.676	-0.2600	-0.62400	1.55e-01	6.34e-04
EUKARYOTIC TRANSLATION INITIATION	114	5.75e-28	0.663	0.2040	0.63100	1.65e-04	2.33e-31
REDUCTION OF CYTOSOLIC CA LEVELS	11	3.08e-02	0.650	-0.5570	-0.33600	1.38e-03	5.39e-02
YAP1 AND WWTR1 TAZ STIMULATED GENE EXPRESSION	11	1.27e-02	0.633	0.1440	0.61600	4.08e-01	4.01e-04
POST CHAPERONIN TUBULIN FOLDING PATHWAY	17	1.31e-04	0.629	0.6290	-0.00378	7.13e-06	9.78e-01
SELENOAMINO ACID METABOLISM	109	2.31e-22	0.629	0.2410	0.58100	1.43e-05	1.04e-25
FORMATION OF TUBULIN FOLDING INTERMEDIATES BY CCT TRIC	19	1.01e-03	0.614	0.5810	0.19900	1.17e-05	1.33e-01
ACTIVATION OF THE MRNA UPON BINDING OF THE CAP BINDING COMPLEX AND EIFS AND SUBSEQUENT BINDING TO 43S	59	2.55e-12	0.609	0.1620	0.58700	3.15e-02	6.12e-15
CGMP EFFECTS	15	8.01e-03	0.604	-0.5630	-0.22000	1.60e-04	1.41e-01
TRANSPORT OF CONNEXONS TO THE PLASMA MEMBRANE	13	7.87e-03	0.604	0.5920	0.12200	2.21e-04	4.48e-01
P75NTR REGULATES AXONOGENESIS	10	9.16e-02	0.592	-0.4920	-0.33000	7.10e-03	7.10e-02
ACTIVATION OF THE AP 1 FAMILY OF TRANSCRIPTION FACTORS	10	5.83e-02	0.587	-0.2020	-0.55100	2.69e-01	2.55e-03
NEUROTOXICITY OF CLOSTRIDIUM TOXINS	10	1.07e-01	0.586	-0.3760	-0.45000	3.97e-02	1.38e-02
HDR THROUGH MMEJ ALT NHEJ	10	1.19e-01	0.572	-0.3550	-0.44900	5.20e-02	1.40e-02
ENDOSOMAL VACUOLAR PATHWAY	11	2.42e-03	0.567	-0.2760	0.49500	1.13e-01	4.45e-03
CLASS C 3 METABOTROPIC GLUTAMATE PHEROMONE RECEPTORS	12	4.96e-02	0.564	-0.2340	-0.51300	1.60e-01	2.09e-03
NONSENSE MEDIATED DECAY NMD	109	2.28e-18	0.564	0.1970	0.52800	3.79e-04	1.54e-21
FORMATION OF ATP BY CHEMIOSMOTIC COUPLING	18	3.08e-03	0.557	0.1250	0.54300	3.59e-01	6.69e-05
COMPLEX I BIOGENESIS	56	6.43e-09	0.556	0.1910	0.52300	1.33e-02	1.32e-11

NITRIC OXIDE STIMULATES GUANYLATE CYCLASE	20	3.83e-03	0.553	-0.5160	-0.19800	6.38e-05	1.25e-01
VOLTAGE GATED POTASSIUM CHANNELS	39	1.38e-04	0.538	-0.3990	-0.36100	1.59e-05	9.68e-05
APOPTOSIS INDUCED DNA FRAGMENTATION	10	1.33e-01	0.529	-0.4740	-0.23500	9.46e-03	1.98e-01
CROSS PRESENTATION OF SOLUBLE EXOGENOUS ANTIGENS ENDOSOMES	46	3.04e-05	0.529	0.3240	0.41800	1.45e-04	9.20e-07
SEROTONIN NEUROTRANSMITTER RELEASE CYCLE	18	9.06e-03	0.528	-0.1550	-0.50400	2.55e-01	2.11e-04
KERATAN SULFATE DEGRADATION	12	1.17e-01	0.524	0.3260	0.41000	5.03e-02	1.39e-02
SEALING OF THE NUCLEAR ENVELOPE NE BY ESCRT III	23	1.14e-03	0.523	0.5120	0.10400	2.12e-05	3.88e-01
REGULATION OF EXPRESSION OF SLITS AND ROBOS	160	6.32e-22	0.521	0.2160	0.47400	2.45e-06	3.81e-25
ACETYLCHOLINE BINDING AND DOWNSTREAM EVENTS	10	8.73e-02	0.505	-0.0816	-0.49800	6.55e-01	6.37e-03
HIGHLY CALCIUM PERMEABLE POSTSYNAPTIC NICOTINIC ACETYLCHOLINE RECEPTORS	10	8.73e-02	0.505	-0.0816	-0.49800	6.55e-01	6.37e-03
RESOLUTION OF D LOOP STRUCTURES THROUGH SYNTHESIS DEPENDENT STRAND ANNEALING SDSA	24	1.00e-02	0.504	-0.2830	-0.41700	1.64e-02	4.06e-04
DSCAM INTERACTIONS	10	1.95e-01	0.501	-0.2740	-0.42000	1.34e-01	2.16e-02
PHASE 0 RAPID DEPOLARISATION	30	4.01e-03	0.495	-0.3090	-0.38600	3.45e-03	2.49e-04
RESPIRATORY ELECTRON TRANSPORT ATP SYNTHESIS BY CHEMIOSMOTIC COUPLING AND HEAT PRODUCTION BY UNCOUPLING PROTEINS	125	3.11e-16	0.492	0.1600	0.46500	2.01e-03	2.80e-19
TRANSLATION	286	4.24e-31	0.489	0.2600	0.41400	3.90e-14	1.70e-33
AUF1 HNRNP D0 BINDS AND DESTABILIZES MRNA	53	4.27e-05	0.489	0.3250	0.36500	4.23e-05	4.35e-06
RESPIRATORY ELECTRON TRANSPORT	102	6.63e-13	0.489	0.1600	0.46200	5.31e-03	7.62e-16
CREB1 PHOSPHORYLATION THROUGH THE ACTIVATION OF ADENYLATE CYCLASE	11	1.95e-01	0.488	-0.3800	-0.30500	2.90e-02	7.97e-02
CYTOSOLIC IRON SULFUR CLUSTER ASSEMBLY	13	1.36e-01	0.487	0.3860	0.29700	1.59e-02	6.37e-02
ASPARTATE AND ASPARAGINE METABOLISM	10	2.34e-01	0.484	-0.3050	-0.37600	9.53e-02	3.96e-02
SYNTHESIS OF PROSTAGLANDINS PG AND THROMBOXANES TX	13	5.22e-02	0.483	0.0845	0.47600	5.98e-01	2.99e-03
STABILIZATION OF P53	54	4.61e-05	0.481	0.3120	0.36600	7.18e-05	3.25e-06
SYNTHESIS OF BILE ACIDS AND BILE SALTS VIA 7ALPHA HYDROXYCHOLESTEROL	12	1.34e-01	0.479	0.2050	0.43300	2.18e-01	9.44e-03
MITOCHONDRIAL TRANSLATION	93	7.64e-09	0.478	0.3130	0.36200	1.92e-07	1.67e-09
WNT5A DEPENDENT INTERNALIZATION OF FZD2 FZD5 AND ROR2	13	4.88e-02	0.474	0.4710	0.05160	3.26e-03	7.47e-01
CASPASE ACTIVATION VIA DEPENDENCE RECEPTORS IN THE ABSENCE OF LIGAND	10	1.78e-01	0.472	-0.1600	-0.44400	3.81e-01	1.51e-02
THE ROLE OF GTSE1 IN G2 M PROGRESSION AFTER G2 CHECKPOINT	66	4.04e-06	0.470	0.3890	0.26400	4.70e-08	2.14e-04

GLUTATHIONE SYNTHESIS AND RECYCLING	11	2.21e-01	0.468	0.2850	0.37100	1.02e-01	3.30e-02

Supplementary Table 2: The Significant Gene Sets Directly Associated with Mental Health and/or Neuroinflammation.

Gene set	Theme	Reference
Class c metabotropic glutamate receptors	**Mental Health**	[61]–[64],[154],[155]
Acetylcholine binding and downstream event		[54]
Highly calcium permeable postsynaptic nicotinic acetylcholine receptors		[55]–[57]
SRP dependent co-translational protein translocation		[65],[156]
P75NTR regulates axonogenesis		[67]–[74]
Serotonin neurotransmitter release cycle		[58]
Activator protein 1 (AP-1) family		[76],[77],[157]
Netrin-1/DCC guidance		[80],[81],[158],[159]
Voltage gated K+ channels		[59],[60]
Nitric oxide stimulates guanylate cyclase, cyclic GMP effects, and reduction of cytosolic Ca		[82]–[84],[86]–[88],[160]
NMD		[104],[105]
Reduction of cytosolic Ca		[161],[162]
Homology-directed repair (HDR), apoptosis-induced DNA fragmentation		[111]–[113]
Translational dysregulation and protein misfolding gene sets		[121]–[124],[163]
Eukaryotic translation initiation		[125],[126]
Keratan sulphate degradation gene set		[127],[128]
Response of EIF2AK4 (GCN2) to amino acid deficiency		[164]
Complex 1 biogenesis		[165]
Regulation of expression of SLITs and ROBOs		[166]–[168]
Yap-1 and Taz (Hippo signalling)		[169],[170]
CREB1 phosphorylation through activation (CRTC1 signalling)		[41]–[44],[171],[172] [45]–[52]
Aspartate and asparagine metabolism		[173]
Formation of ATP chemiosmotic coupling, *Complex I biogenesis*, *Respiratory electron transport* and *Mitochondrial translation*		[114],[115],[119],[174]
**Gene set**	**Theme**	**Reference**
Activator protein 1 (AP-1) family	**Neuroinflammation**	[78]
Nitric oxide stimulates guanylate cyclase, *Cyclic GMP effects*, and *Reduction of cytosolic Ca*		[92]–[94]
ATP metabolism-related gene sets		[120],[175]
Response of EIF2AK4 (GCN2) to amino acid deficiency		[176]
Regulation of expression of SLITs and ROBOs		[177]
Metabotropic glutamate receptors		[178]
Yap-1 and Taz (Hippo signalling)		[179]
Stabilization of p 53		[180]
Caspase activation		[181]
Aspartate and asparagine metabolism		[182]
Cytoprotection by HMXO1		[183]
Regulation of HMXO1 expression and activity		[184],[185]
Detoxification of reactive oxygen species		[186]
Sealing of the nuclear envelope by ESCRT-III		[187]
